# A proposal to grade the severity of early allograft dysfunction after liver transplantation

**DOI:** 10.1590/S1679-45082013000100006

**Published:** 2013

**Authors:** Paolo Salvalaggio, Rogerio Carballo Afonso, Guilherme Felga, Ben-Hur Ferraz-Neto

**Affiliations:** 1 Unidade de Transplante de Fígado, Hospital Israelita Albert Einstein, São Paulo, SP, Brazil

**Keywords:** Liver transplantation, Postoperative complications, Reoperation, Graft survival

## Abstract

**Objective:**

To propose a grading system for early hepatic graft dysfunction.

**Methods:**

A retrospective study from a single transplant center. Recipients of liver transplants from deceased donors, transplanted under the MELD system were included. Early graft dysfunction was defined by Olthoff criteria. Multiple cut-off points of post-transplant laboratory tests were used to create a grading system for early graft dysfunction. The primary outcome was 6-months grafts survival.

**Results:**

The peak of aminotransferases during the first postoperative week correlated with graft loss. The recipients were divided into mild (aminotransferase peak >2,000IU/mL, but <3,000IU/mL); moderate (aminotransferase peak >3,000IU/mL); and severe (aminotransferase peak >3,000IU/mL + International Normalized Ratio ≥1.6 and/or bilirubin ≥ 10mg/dL in the 7^th^ postoperative day) early allograft dysfunction. Moderate and severe early dysfunctions were independent risk factors for graft loss. Patients with mild early dysfunction presented with graft and patient survival comparable to those without graft dysfunction. However, those with moderate early graft dysfunction showed worse graft survival than those who had no graft dysfunction. Patients with severe early dysfunction had graft and patient survival rates worse than those of any other groups.

**Conclusion:**

Early graft dysfunction can be graded by a simple and reliable criteria based on the peak of aminotransferases during the first postoperative week. The severity of the early graft dysfunction is an independent risk factor for allograft loss. Patients with moderate early dysfunction showed worsening of graft survival. Recipients with severe dysfunction had a significantly worse prognosis for graft and patient survival.

## RESUMO

**Objetivo:**

Propor um sistema de graduação para a disfunção precoce do enxerto hepático.

**Métodos:**

Estudo retrospectivo de um único centro transplantador. Foram incluídos receptores de transplante hepático por doador falecido transplantados pelo sistema MELD. A disfunção precoce do enxerto foi definida segundo os critérios de Olthoff. Diversos pontos de corte para testes de laboratório pós-transplante foram utilizados para criar um sistema de graduação da disfunção precoce do enxerto. O principal desfecho foi a perda do enxerto aos 6 meses.

**Resultados:**

O pico de aminotransferases durante a primeira semana pós-operatória se correlacionou com a perda do enxerto. Os receptores foram divididos em disfunção precoce do enxerto leve (pico de aminotransferases >2.000UI/mL, mas <3.000UI/mL); moderada (pico de aminotransferases >3.000 UI/mL); e grave (pico de aminotransferases >3.000UI/mL + *International Normalized Ratio ≥*1,6 e/ou bilirrubina ≥10mg/dL no 7° dia pós-operatório). Disfunções precoces moderada e grave, foram fatores de risco independentes para a perda do enxerto. Pacientes com disfunção precoce leve apresentaram sobrevida do enxerto e do paciente comparável àqueles sem disfunção do enxerto. Contudo, aqueles com disfunção precoce moderada tiveram pior sobrevida do enxerto comparada aos que não tiveram disfunção do enxerto. Pacientes com disfunção precoce grave tiveram sobrevida do enxerto e do paciente pior do que os outros grupos.

**Conclusão:**

Disfunção precoce do enxerto pode ser graduada por meio de um critério simples e confiável, baseado no pico de aminotransferases durante a primeira semana de pós-operatório. A gravidade da disfunção precoce do enxerto é um fator de risco independente para a perda do enxerto. Pacientes com disfunção precoce moderada tiveram pior sobrevida do enxerto. Receptores com disfunção precoce grave tiveram um prognóstico significativamente pior de sobrevida do enxerto e do paciente.

## INTRODUCTION

Advances in surgery, anesthesia, immunosuppression and medical care have contributed to the current success of liver transplantation across the globe^(1)^. The modern transplant physician deals not only with extremely sick transplant candidates and non-ideal donors, but also with small financial margins and growing pressure of regulatory agencies that measure transplant outcomes^(2–8)^. Recently, there has been a growing interest in the development of benchmarks that correlate initial graft function and post-transplant outcomes^(9–13)^.

Early allograft dysfunction (EAD) is a clinical entity which might reflect donor, recipient and transplant characteristics that impact early graft function and could be utilized as a transplant benchmark. Earlier single-center studies have tried to define EAD in the pre-Model for End-Stage Liver Disease (MELD)^(14–17)^. Other terms such as “poor initial function” or “graft dysfunction with or without inclusion of primary non-function and vascular complications” have also been proposed^(12,13)^. Recently, in the MELD era, EAD has been defined in those patients with a substantial elevation of aminotransferases during the first postoperative week, or in those who are significantly jaundiced or have a coagulation disorder on the 7th postoperative day. The criterion chosen was based on prior studies and expert opinions of large transplant centers in United States. Importantly, this criterion highly correlated with 6-month patient and graft survival^(10)^.

In Brazil, EAD impacts our daily clinical practice. It is our clinical impression that some patients who have EAD recover extremely fast and do well. On the other extreme, EAD might correlate with similar donor, recipient and surgical factors that were described in recipients with primary non-function (PNF)^(18–21)^. One could postulate that PNF might be the most severe grade of EAD.

A potential gap in previous studies of EAD is the inability to differentiate the severity of this entity. We hypothesize that patients with EAD could be better characterized in a wide clinical spectrum instead of in a single group that behaves uniformly. We strongly believe that a grading system for EAD could assist the clinician in making prompt decisions regarding graft viability, potential retransplantation and eventually innovative interventions that would allow early graft rescue. We designed this study to create a grading system for EAD.

## OBJECTIVE

To propose a grading system for early allograft dysfunction.

## METHODS

This is a retrospective cohort study that was initially conducted by including data from all recipients of liver transplant performed at Hospital Israelita Albert Einstein (HIAE) from July 1^st^, 2005 through June 30^th^, 2010. Data were drawn from the liver transplant database and electronic medical records of our hospital. For the present study, inclusion was restricted to adult patients (≥18 years of age) who were candidates for the first deceased donor liver transplantation. Patients with liver-kidney transplants and partial grafts were included. Those with vascular complications and PNF within the first postoperative week were excluded. PNF was described according to the definition of the United Network for Organ Sharing (UNOS), within 7 days of implantation, as defined by aspartate aminotransferase (AST) ≥3,000 and one or both of the following: International Normalized Ratio (INR) ≥2.5 or acidosis, defined as having an arterial pH ≤7.30 or venous pH of 7.25 and/or lactate ≥4mMol/L^(22)^.

### EAD definition and classification

We defined EAD in patients who had: (1) bilirubin ≥10mg/dL on postoperative day 7; and/or (2) INR ≥1.6 on postoperative day 7; and/or (3) aminotransferase peak (alanine aminotransferase – ALT – or AST) >2,000IU/mL within the first 7 postoperative days^(11)^.

Searching for a valid classification, we first used different cut-off points of these three laboratory tests with and/or without adding other variables such as PNF, encephalopathy, acidosis, and the ability to clear lactic acid. For each quartile of distribution of the results of bilirubin and INR at the 7th day or the peak of aminotransferases in the first week, we performed concordance statistics (c-statistic) with the risk of 6-month allograft loss.

A c-statistic between 0.8 and 0.9 was interpreted as having excellent discriminative ability. A test with a c-statistic of 0.65 and higher was interpreted as potentially useful tool. A test with a c-statistic <0.6 was judged not useful^(10,12,23)^. Relative risks (RRs) with 95% confidence intervals (95%CI) were calculated as the cumulative incidence of mortality within 6 months among those with EAD divided by the incidence of 6-month mortality among those without EAD.

We constructed the ROC curves with different combinations of levels of aminotransferases, degree of cholestasis, significance of coagulation disorders and the variables here described. We then picked the grading system which had the best c-statistic at the same time that would be easy to use and practical for the clinician at the bedside, who is attempting to calculate the risk of graft loss based on the severity of EAD.

### Groups

To validate the correlation of EAD severity and post-transplant outcomes, we next divided the study population into four groups: no-EAD, mild EAD, moderate EAD, and severe EAD. Patients who did not have EAD were included in the no-EAD group (reference group). Mild EAD was defined in those who had peaks of aminotransferases during the initial postoperative week >2,000IU/mL but <3,000IU/mL. Those with moderate EAD had a peak of aminotransferases during the initial postoperative week ≥3,000IU/mL, without any severe alteration of bilirubin (≥10mg/dL on 7^th^ postoperative day) or INR ≥1.6 on 7^th^ postoperative day. Patients who had a peak of aminotransferases ≥3,000IU/mL in the first postoperative week, in association with bilirubin ≥10mg/dL and/or INR≥1.6 by the 7th postoperative day, were included in the severe EAD group.

### Severity of EAD as a risk factor for graft loss

In order to test the proper correlation of EAD with allograft loss we performed a univariate analysis utilizing 6-month graft loss as endpoint. Those factors that had p≤0.2 were entered into a multivariate analysis. In order to test whether different grades of EAD could independently contribute for allograft loss, we employed a Cox model.

### Covariates and other definitions

Covariates included gender, age, race, ethnicity, blood type, height, weight, body mass index (BMI), cause of liver failure (viral hepatitis, hepatocellular carcinoma – HCC – and other causes), local *versus* regional *versus* national graft, split *versus* full grafts, kidney cotransplantation, donor age, gender, height, weight, BMI, donor risk index (DRI), blood transfusion and cold ischemiatime (CIT)^(24)^. We utilized definitions of allograft loss and patient death equal to those found in the Organ Procurement and Transplant Network (OPTN) registry. The biological MELD at the time of the transplant (or the last score available) was calculated as previously published^(25)^. Donation after cardiac death (DCD) is not present in this series. Due to the variety of races in the country, the races of the donors are not reported in the database^(26)^. To calculate the DRI we set DCD scores to zero and imputed race scores to 0.15 (average between minimum and maximum allowed scores).

### Statistical analysis

Comparisons between rates for demographic, clinical, and geographic strata for the two eras were performed using the χ^2^ test to examine qualitative variables and Analysis of Variance (ANOVA) to study quantitative variables. Kaplan-Meier curves were drawn depicting the post-transplant patient and graft survival differences of patients by group. The log-rank test was used to determine if there was a significant difference in the curves. Missing data on the characteristics examined was categorized as “other” or “unknown” or excluded from analysis (in most circumstances), depending on the frequency of missing data for the given characteristic. No imputation technique was used. An alpha level of 0.05 was used for all significance tests. Analyses were performed using SAS v.9.2 (SAS Institute, Cary, NC).

This study was approved by the Research Ethics Committee of the Institution under number CAAE 079721129.0000.0071.

## RESULTS

### Number of patients included in the study

During the period of study, 458 liver transplants were performed at our unit. After we applied the inclusion and exclusion criteria, 325 patients formed the population of this study.

### Classification of EAD

**Figure 1:**
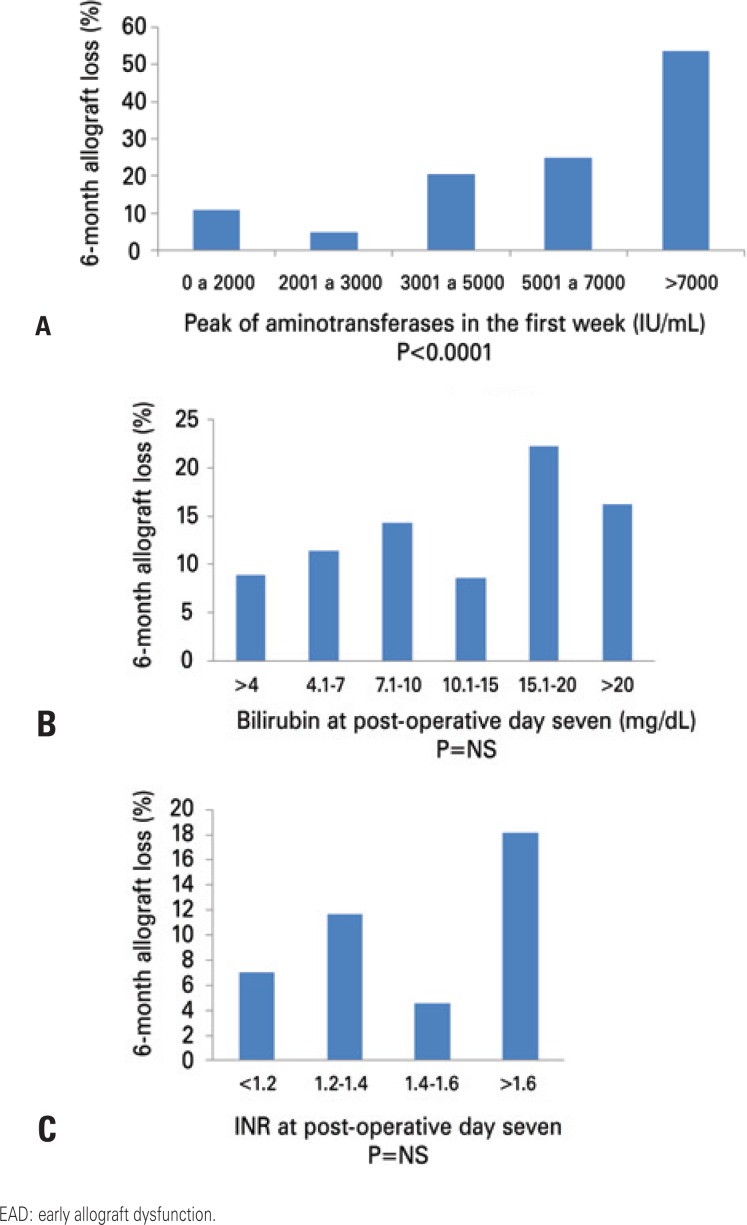
Relationship of aminotransferase peak in the first week (A), bilirubin (B) and INR (C) at day 7 with 6-month allograft loss

Figure 1 shows the correlation of variables included in the grading system. When taken individually, INR and bilirubin did not present a strong correlation with graft loss. However, when we observed those with aminotransferases >2,000IU/mL within the first week, we found a strong correlation between the peak of aminotransferases and graft loss. We then tested a variety of combinations of different cut-off points to discriminate allograft loss. The current grading system had a c-statistic of 0.68. Encephalopathy, acidosis (using pH as surrogate) or lactic acid clearance did not increase the c-statistic (c<0.6). We have also tried to create two to four EAD groups, but finally chose to limit the analysis only to three groups, based primarily on the peak of aminotransferases and in combination with the presence of an abnormal INR (≥1.6) or bilirubin level (≥10mg/dL) at the 7 th postoperative day.

### Clinical characteristics of the study cohort and donor demographics

The demographics of the transplant recipients are depicted in table 1. When we compared recipients with mild, moderate and severe EAD with those without EAD we found no differences among the groups. Nonetheless, multiple donor characteristics were found to be different among the groups, including gender, height, weight, BMI and type of graft (Table 2).

**Table 1:** Demographics of transplant recipients included in the study

Variable		No EAD n=142 (43.7%)	Mild EAD n=93 (28.6%)	Moderate EAD n=58 (17.8%)	Severe EAD n=32 (9.8%)	p value
Gender of recipient						
	Male	103 (72.5)	69 (74.2)	39 (67.2)	18 (56.3)	0.230
Age of recipients (years) average±SD		52.7±11.6	51.7±12.4	48.5±11.3	53.6±12	0.108
	18-39	17 (12)	16 (17.2)	14 (24.1)	5 (15.6)	0.194
	40-49	33 (23.2)	16 (17.2)	16 (27.6)	6 (18.8)	
	50-59	53 (37.3)	38 (40.9)	20 (34.5)	9 (28.1)	
	>60	39 (27.5)	23 (24.7)	8 (13.8)	12 (37.5)	
Race of recipient						
	White	119 (86.2)	77 (85.6)	44 (78.6)	22 (71)	0.303
	Brown	17 (12.3)	12 (13.3)	10 (17.9)	7 (22.6)	
	Others	2 (1.5)	1 (1.1)	2 (3.6)	2 (6.5)	
Average height (cm)		169.7±9.2	168.5±8.9	167.9±8.7	166.2±11	0.236
Average weight (kg)		77.8±16.0	74.8±13.8	78.2±17.7	77.4±20.5	0.503
Average BMI		26.9±4.7	26.3±4.1	27.6±5.3	27.7±5.1	0.288
Blood type of recipient						
	A	67 (47.2)	42 (45.2)	22 (37.9)	10 (31.3)	0.454
	B	15 (10.6)	13 (14)	8 (13.8)	5 (15.6)	
	AB	3 (2.1)	4 (4.3)	4 (6.9)	0 (0)	
	O	57 (40.1)	34 (36.6)	24 (41.4)	17 (53.1)	
Primary diagnosis						
	Hepatocarcinoma	53 (37.3)	30 (32.3)	19 (32.8)	10 (31.3)	0.817
	Hepatitis B Virus	10 (7)	6 (6.5)	1 (1.7)	3 (9.4)	0.850
	Hepatitis C Virus	66 (46.5)	37 (39.8)	29 (50)	13 (40.6)	
	Alcohol	23 (16.2)	16 (17.2)	6 (10.3)	3 (9.4)	
	Acute liver failure	8 (5.6)	8 (8.6)	5 (8.6)	4 (12.5)	
	Cryptogenic	14 (9.9)	10 (10.8)	5 (8.6)	3 (9.4)	
	Other	21 (14.8)	16 (17.2)	12 (20.7)	6 (18.8)	
Pre-transplant characteristics						
	Dialysis	10 (9.8)	4 (6.4)	5 (11.9)	2 (8.7)	0.825
	MELD at transplantation	21.6±9.9	21.8±10.8	21.0±11.7	18.1±9.0	0.332
	Previous surgery	27 (19)	16 (17.2)	7 (12.1)	6 (18.8)	0.693

EAD: early allograft dysfunction; SD: standard deviation; BMI: body mass index; MELD: Model for End-Stage Liver Disease.

**Table 2:** Donor demographics and transplant characteristics

Variable		No EAD n=142 (43.7%)	Mild EAD n=93 (28.6%)	Moderate EAD n=58 (17.8%)	Severe EAD n=32 (9.8%)	p value
Gender of donor						
	Male (%)	72 (51.8)	55 (59.8)	29 (50.9)	26 (81.3)	0.0157
Age of donor (years) average±SD		42.2±19.3	43.6±17.0	45.6±16.0	45.1±12.1	0.6049
	0-45 (%)	72 (52.2)	47 (52.2)	25 (44.6)	15 (48.4)	0.2392
	>45 (%)	66 (47.8)	43 (47.8)	31 (55.4)	16 (51.6)	
Height (cm)		162.9±16.5	167.1±13.5	169.4±9	171.4±9.5	0.0021
Weight (kg)		66.7±18	72.2±15.6	73.8±13.4	79.2±14.6	0.0003
BMI		24.4±4.4	25.5±4.1	25.6±3.5	27±5.1	0.0142
Origin of the graft (%)						
	Local	107 (81.7)	71 (78.9)	41 (74.6)	23 (74.2)	0.9485
	Regional	6 (4.6)	5 (5.6)	4 (7.3)	2 (6.5)	
	National	18 (13.7)	14 (15.6)	10 (18.2)	6 (19.4)	
Cause of donor death						
	Cerebrovascular accident	74 (56.5)	53 (58.9)	32 (58.2)	18 (58.1)	0.9989
	Trauma	47 (35.9)	31 (34.4)	20 (36.4)	11 (35.5)	
	Anoxia	8 (6.1)	4 (4.4)	2 (3.6)	0 (0)	
	Others	2 (1.5)	2 (2.2)	1 (1.8)	2 (6.5)	
Type of graft						
	Split grafts	5 (3.5)	12 (12.9)	7 (12.1)	1 (3.1)	0.0984
	Domino donor	7 (4.9)	0 (0)	1 (1.7)	0 (0)	
	Deceased donor	128 (90.1)	78 (83.9)	48 (82.8)	30 (93.8)	
	Liver-kidney	2 (1.4)	3 (3.2)	2 (3.5)	1 (3.1)	
CIT (hours)		9.1±2.8	8.3±2.1	8.9±2.7	9.3±3.1	0.1687
DRI		1.9±0.5	1.9±0.5	2.1±0.7	1.9±0.4	0.2058
Transfusion of RBCs (units)		1.6±2.0	2.3±3.0	2.1±3.0	2.0±2.5	0.1794

EAD: early allograft dysfunction; SD: standard deviation; BMI: body mass index; CIT: cold ischemia time; DRI: Donor Risk Index; RBC: red blood cells.

### Risk factors for allograft loss

Table 3 shows donor, recipient and transplant characteristics that could be related to 6-month graft loss. The univariate analysis pointed to EAD, race of recipient, height of recipient, CIT and multiple transfusions as potential risk factors for allograft loss. However, a Cox analysis isolated only race, height and gender of the recipient, CIT, multiple blood transfusions and severity of EAD as risk factors for graft loss.

**Table 3:** Proportional hazards of graft loss (multivariate analysis by Cox regression)

Variable		Analysis univariate p-value	Multivariate analysis	
			Adjusted relative risk	p value
			(95%CI)	
EAD		<0.0001		< 0.0001
	No EAD			
	Mild EAD		0.51 (0.23-1.14)	
	Moderate EAD		1.51 (0.75-3.06)	
	Severe EAD		3.64 (1.80-7.34)	
Gender of recipient		0.0045		0.7264
	Female		0.88 (0.43-1.80)	
Age of recipient (continuous)		0.1709	0.98 (0.96-1.004)	0.1031
Age of recipient (categorized)		0.1181		0.0845
	>45		0.60 (0.34-1.07)	
Race of recipient		<0.0001		0.0067
	White/Brown			
	Others		3.93 (1.46-10.56)	
Height of recipient		0.0067		0.0402
	≤165cm		1.77 (1.03-3.05)	
Weight of recipient		0.0890		
BMI of recipient		0.3519		
Blood type of recipient		0.7377		
Hepatocarcinoma		0.2123		
Primary diagnosis		0.1179		0.8882
Pre-transplant characteristics				
	Dialysis	0.6194		
	MELD at transplantation	0.1074	1.02 (0.99-1.04)	0.1336
Previous surgery		0.8760		
Gender of donor		0.7260		
Age of donor (continuous)		0.8790		
Age of donor (categorized)		0.2635		
Height of donor		0.1901	0.99 (0.97-1.01)	0.1750
Weight of donor		0.2152		
BMI of donor		0.3749		
Imported graft		0.9414		
Cause of donor death: CVA		0.8465		
Split and domino grafts		0.4510		
CIT		0.0082		0.0010
	≤9 hours		3.15 (1.59-6.24)	
DRI		0.9088		
Transfusion of RBC (units)		0.0098	1.11 (1.03-1.19)	0.0071

95%CI: 95% confidence interval; EAD: early allograft dysfunction; BMI: body mass index; MELD: Model for End-Stage Liver Disease; CVA: cerebral vascular accident; CIT: cold ischemia time; DRI: Donor Risk Index; RBC: red blood cells.

### Post-transplant outcomes

**Figure 2:**
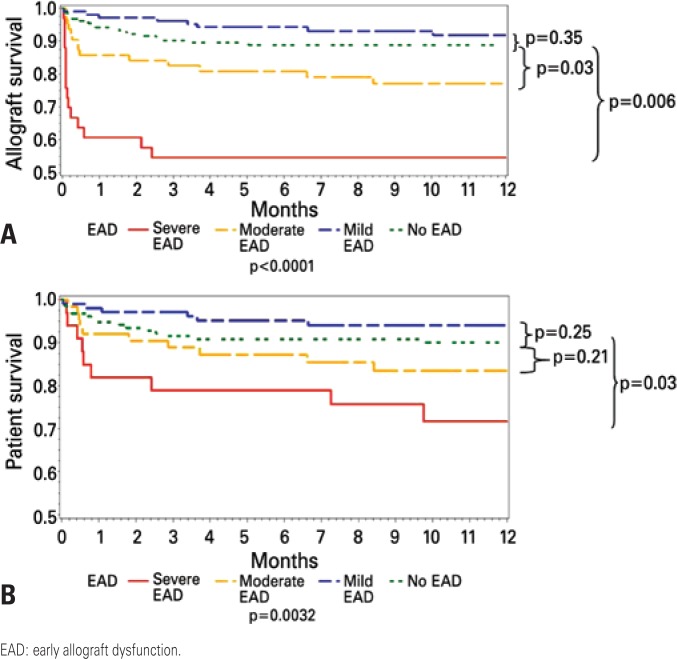
Non-adjusted graft (A) and patient (B) survival according to severity of EAD

Patients with severe EAD were retransplanted more often than all other groups. Most of the retransplants were performed early due to poor function. Patients with EAD had worse graft (Figure 2A) and patient survival (Figure 2B) than those without EAD. Those with mild EAD had 1-year patient (94%) and graft (91.8%) survival comparable to those without EAD (90 and 88.9%, respectively). Those with moderate EAD had worse 1-year graft survival (77.2%) than those without EAD (p=0.03) and those with mild EAD (p=0.006). Patients with moderate EAD had 1-year patient survival (83.5%) comparable to those without EAD and worse 1-year patient survival than those with mild EAD (p=0.03). Those with severe EAD had a significant worsening in 1-year grafts (54.6%) and patients (71.7%) than all other groups (p<0.001).

## DISCUSSION

Despite of the relationship between EAD and 6-month survival, there is still a need to quickly separate patients with EAD who will rapidly recover from those who do not do well. Therefore, we designed this study to create a grading system for EAD.

We initially looked at different cut-off points of the postoperative liver tests. The main finding of our study is the demonstration that the peak of aminotransferases correlates significantly with 6-month patient and graft survival and can, therefore, be utilized to grade EAD. There was a random distribution of INR and a lack of correlation of bilirubin levels with post-transplant outcomes. The inclusion of other variables did not seem to increase the discriminatory ability to predict post-transplant outcomes. At the same time, by adding more variables, we increased subjectivity (such as encephalopathy) or made the grading system complicated for daily clinical use (such as clearance of lactic acid).

One interesting finding of our study is a higher rate of EAD in our population in comparison to prior reports^(10,12)^. Indeed, the significance of the problem in our clinical practice was the main reason we assigned a research group to focus on EAD. We have not yet identified the details behind this discrepancy. Most likely there is a correlation with donor quality and management. In Brazil, hospitals and intensive care units still lack resources to properly sustain and manage the brain-dead donor. Moreover, we found DRIs significantly higher on this case selection than in most liver transplant reports, which might signal that the quality of our donor population could indeed be different from that of European and North American transplant centers^(24)^. Other potential explanations include variability in donor acceptance criteria, organ preservation, surgical and anesthesia techniques that are current areas of research and quality improvement initiatives of our group.

It is important to highlight that we started with a different question and a hypothesis that was driven by clinical findings in dealing daily with patients with EAD. We were not attempting to create a new definition of EAD or to validate prior studies. In our point of view, the EAD definition is reliable and appropriate^(10)^. Thus, our findings refine the most current definition of EAD. We innovate by proposing how to measure EAD. Thus, future clinical and translational studies of EAD will now have two options in measuring EAD. Researchers can opt to utilize EAD as a discrete nominal variable (yes *versus* no) or as a continuous variable. It will be critical for other groups to validate our findings or to improve EAD measurement methods with better scales or other systems.

Our grading system based mainly on the peak of aminotransferases is intuitive, easily reproducible, and has a good relationship with post-transplant survival. However, we were puzzled by the results found in the group of patients with mild EAD. Kaplan Meier survival curves were slightly superior (but not statistically different) than those of patients without EAD. Thus, the grading system allowed us to identify two subpopulations that are at higher risk of poor outcomes. Patients with moderate EAD had a higher risk for graft loss. Those with severe EAD had a significantly higher odds ratio of graft loss and mortality. These two groups need not only better clinical support, but also potential allocation policy changes and quicker decisions regarding retransplantation. Indeed those patients with severe EAD are the main contributors for a poor post-transplant outcome at our center. This subpopulation has a higher retransplantation rate. Our findings corroborate our hypothesis that patients with EAD behave differently, which is probably a combination of the severity of ischemia-reperfusion injury of the new liver graft, associated with the comorbidities and general health status of the recipient and perioperative management. We will now concentrate our efforts on improving future outcomes, selection and management in these patients. New technologies that will allow earlier diagnosis, graft protection, or reversibility of EAD will be paramount in the current environment of liver transplantation of meager (and never sufficient number of) donors, sick recipients and stiff regulations^(5,8,9,11)^. The impact of ischemiareperfusion, immunosuppression, liver regeneration, genomics, proteomics and molecular pathways on EAD should be further investigated, and novel technologies, such as the LiMax assay, should be further explored^(11)^.

We searched for risk factors for allograft loss and whether different degrees of EAD would increase the risk of allograft loss. The factors that were relevant, such as prolonged CIT and utilization of blood transfusion, have been previously described^(27–29)^. For those factors in which the surgeon can intervene by reducing CIT or blood loss, a concomitant reduction of EAD severity might be possible. However, other important characteristics, such as the MELD score of the recipient, pre-transplant dialysis and donor age have been previously pointed as risk factors for EAD and graft loss, but were not found to be relevant in our analysis.

One major focus of the liver transplant community has spun around the utilization of expanded criteria donors (ECDs)^(27–33)^. Most characteristics that were previously included in the ECD definition were not found to be risk factors for allograft loss in our analysis. Thus, we suggest a different approach to address the repercussions of the ECDs in post-transplant outcomes. An initial step might be to separate those recipients who do well from those who will have a poor outcome. For this purpose, classifications, scales and grading systems (in similarity with the EAD grading system) might be important contributions. Prognostic models, economic studies and descriptions of complications related to EAD and ECDs are certainly needed. We hypothesize that EAD in liver transplantation might mirror what has been described in kidney allografts that work poorly but do not survive as well in the long-range as do those with initial good function^(34)^. Long-term follow-up of our cohort should contribute to answering this question.

Our study has several limitations. First, those inherent to single-center retrospective studies. There was also a limitation of the size of our population of study, which was certainly less than ideal for a clean statistical analysis, but at the same time was comparable to recent studies of EAD^(10,12)^. Second, we started with a set definition of EAD, created a classification and looked into the outcomes of the cohort. We recognize that this is not ideal, but, based on sample size, it is an initial approach. Finally, the grading system has a c-statistic that is acceptable, but not ideal. This compares with several clinical tools currently in use in liver transplantation and surgery^(23,35–37)^. It is certainly important that futures studies test and validate this classification.

## CONCLUSION

In summary, we created a grading system for EAD. Patients with severe EAD had significantly worse patient and graft survival than any other group of our study. Patients with moderate EAD had worse graft survival when compared to patients without EAD. EAD is an independent risk factor for allograft loss. Future studies should search for early markers of EAD and interventions that could minimize or reverse graft damage and loss.
